# A safety comparison of heparin and argatroban anticoagulation in veno‐venous extracorporeal membrane oxygenation with a focus on bleeding

**DOI:** 10.1111/tme.13102

**Published:** 2024-10-07

**Authors:** Filip Burša, Jan Máca, Jiří Sagan, Peter Sklienka, Simona Němcová, Zuzana Kučerová, Tereza Romanová, Ondřej Jor, Adéla Kondé, Jaroslav Janošek, Michal Frelich

**Affiliations:** ^1^ Department of Anaesthetics, Resuscitation and Intensive Care Medicine University Hospital Ostrava Ostrava Czech Republic; ^2^ Institute of Physiology and Pathophysiology, Faculty of Medicine University of Ostrava Ostrava Czech Republic; ^3^ Department of Infectious Diseases University Hospital Ostrava Ostrava Czech Republic; ^4^ Department of Applied Mathematics, Faculty of Electrical Engineering and Computer Science VSB– Technical University of Ostrava Ostrava Czech Republic; ^5^ Department of the Deputy Director for Science, Research and Education University Hospital Ostrava Ostrava Czech Republic; ^6^ Center for Health Research, Faculty of Medicine University of Ostrava Ostrava Czech Republic

**Keywords:** anticoagulation, argatroban, bleeding, extracorporeal membrane oxygenation (ECMO), heparin

## Abstract

**Background:**

Anticoagulation during extracorporeal membrane oxygenation (ECMO) might still lead to severe bleeding complications. Heparin is the most frequently used anticoagulant, but novel drugs could be promising. Argatroban is a new alternative to heparin. To date, no robust studies have confirmed the clear superiority of argatroban (AG) over heparin, although it has some advantages and may be safer.

**Study Design and Methods:**

An observational study was conducted in all adult veno‐venous ECMO patients with COVID‐19‐associated acute respiratory distress syndrome admitted to the University Hospital Ostrava (*n* = 63). They were anticoagulated with heparin in the first period and with AG in the second period, targeting the same activated partial thromboplastin time (aPTT; 45–60 s). Bleeding complications requiring transfusion and life‐threatening bleeding events were evaluated. The primary objective was to compare heparin and AG in terms of bleeding, transfusion requirements and mortality‐related bleeding.

**Results:**

The total time on ECMO per patient was 16 days with an in‐hospital mortality of 55.6%. The red blood cell consumption in the AG group (median 2.7 transfusions/week) was significantly lower than in the heparin group (median 4.2 transfusions/week, *p* = 0.011). Life‐threatening bleeding complications were higher in the heparin group compared to the AG group (35.7% vs. 10.2%, *p* = 0.035), and mortality‐related bleeding complications were also higher in the heparin group (21.4% vs. 2.0%, *p* = 0.032).

**Discussion:**

Argatroban is an interesting alternative to heparin with less bleeding, less need for red blood cell transfusions and improved safety of ECMO with less mortality‐related bleeding.

## 1 INTRODUCTION

Veno‐venous extracorporeal membrane oxygenation (VV ECMO) is a life‐saving support method for patients with respiratory failure.^1^ It is, however, still associated with significant risks, and mortality of ECMO patients is high.^2^ While bleeding complications constitute a major challenge in patient management and can be life‐threatening, oxygenator thrombosis usually does not pose such a high risk as the oxygenator function typically deteriorates gradually, which allows timely elective oxygenator exchange.^3^, ^4^ Therefore, bleeding complications are a more serious problem than thrombotic complications and the trend is to reduce anticoagulation.^5^ In addition, bleeding complications are a challenge in terms of care and management and increase the cost of treatment. Unfractionated heparin (UFH) is the most commonly used ECMO anticoagulant^6^; many ECMO centers, however, have introduced the use of novel drugs potentially offering improved anticoagulation management. Of these drugs, argatroban (AG), a direct thrombin inhibitor (DTI), is one of the most promising ones, with many possible advantages over UFH.^7^

In this study, we aimed to evaluate the safety of anticoagulation with UFH versus AG from the perspective, of (i) bleeding complications requiring transfusion (ii) life‐threatening bleeding events, and (iii) bleeding‐related mortality in VV ECMO patients with COVID‐19‐associated acute respiratory distress syndrome (CARDS).

## 2 STUDY DESIGN AND METHODS

This observation monocentric study was performed in the Intensive Care Department of the University Hospital Ostrava, Czech Republic. The study was approved by the Ethics Committee of the University Hospital Ostrava. Informed consent was not needed. The trial has been registered on ClinicalTrials.gov, NCT06038682, registered 15 August 2023–retrospectively registered, https://classic.clinicaltrials.gov/ct2/show/NCT06038682. Adult patients with acute respiratory distress syndrome (ARDS), defined according to the Berlin definition,^8^ infected with SARS‐CoV‐2 and hospitalised in the intensive care unit using VV‐ECMO between April 2020 and July 2023 were enrolled. All ECMO support was provided by systems using centrifugal pumps and coated tubing according to the manufacturer. X lung (Xenios, Fresenius Medical Care), PLS (Rotaflow, Getinge) and HLS (Cardiohelp, Getinge) systems were used. Individual systems were used according to their availability at the time of cannulation. The patients were anticoagulated with UFH from April 2020 to February 2021; from February 2021 onwards, anticoagulation was switched to AG. There was no other difference in the general management of CARDS patients between the two groups. ECMO started with a bolus of heparin (50–70 U/kg plus 5000 U in priming volume), which was followed by UFH (5–10 U/kg/hour) or AG (5–10 μg/kg/hour) anticoagulation with targets of aPTT 45–60 s. aPTT was measured four times per day (6, 12, 18 and 24 o'clock). Anticoagulation dosage was adjusted according to aPTT levels and selected clinical situations such as the presence of coagulopathy, thrombocytopenia, post‐operative status and so forth. The number of blood transfusions, plasma and platelets administered was monitored. Life‐threatening bleeding was defined as the administration of more than six blood transfusions in 24 h. Mortality‐related bleeding was defined as clinically refractory profuse bleeding with hemodynamic failure or bleeding leading directly to death, such as intracranial haemorrhage. Demographic data, aPTT levels, oxygenator changes and life‐threatening oxygenator thromboses (critical sudden drop in VV ECMO efficacy leading to hypoxemia and patient instability) were compared between the groups. The first 12 h of treatment were not included in the analysis because of the initial heparin exposure and its potential effects on coagulation test results. Pathromtin® SL (Siemens Healthcare Diagnostics; Sysmex CS‐5100 analyser) was used for aPTT measurement. Tests were performed according to the manufacturer's instructions. During the bleeding event, best clinical practice was followed and treatment was administered according to the treating physician and the patient continued to be monitored. Oxygenator function and laboratory coagulation parameters were monitored daily according to protocol. If, during the oxygenation test, the post‐oxygenator PO_2_ dropped below approximately 25 kPa, or if low fibrinogen levels were repeatedly measured (below 2 g/L) despite initial fibrinogen supplementation, an elective oxygenator exchange was performed.

Numerical variables are presented as medians, minimums and maximums; categorical variables are presented as absolute and relative frequencies (%). Between‐group differences were assessed using the Mann–Whitney test, the Chi‐square test of independence for contingency tables, or Fisher's exact test as appropriate. Intra‐individual variability of patients in aPTT was evaluated using the robust coefficient of variation (RBV). Multiple boxplots or multiple boxplots combined with the kernel density plots were used to visualise the comparison of groups in selected variables. The significance level was set to 0.05 and the statistical analysis was performed in the R software (version 4.3.1).

## 3 RESULTS

Description of the study group and outcomes are described in Table 1.

**TABLE 1:** Description of the study group and outcomes.

	Median (Min; Max) or *n* (%)			
	Total (*n* = 63)	AG group (*n* = 49)	UFH group (*n* = 14)	*p*
Demographic data				
Sex (male)	38 (60.3)	32 (65.3)	6 (42.9)	0.228
Age (years)	50 (27; 69)	48 (27; 65)	53 (31; 69)	0.267
Weight (kg)	93 (70; 158)	93 (70; 145)	95 (72; 158)	0.813
Body‐mass index (kg/m^2^)	31.2 (20.9; 58.1)	31.3 (20.9; 50.3)	31.0 (24.3; 58.1)	0.953
COVID‐19 variant				0.002
Alpha	40 (64)	26 (53)	14 (100)	
Delta	21 (33)	21 (43)	0 (0)	
Omicron	2 (3)	2 (4)	0 (0)	
APACHE II Score	32 (14; 44)	33 (14; 44)	30 (26; 43)	0.557

Treatment and outcomes				
Circuit type				0.891
Xlung	36 (57)	29 (59)	7 (50)	
PLS	4 (6)	3 (6)	1 (7)	
HLS	23 (37)	17 (35)	6 (43)	
Time on ECMO per patient (days)	16 (1; 124)	18 (1; 124)	12 (5; 28)	0.030
Successful weaning from ECMO	30 (47.6)	26 (53.1)	4 (28.6)	0.189
Life‐threatening bleeding complications	10 (15.9)	5 (10.2)	5 (35.7)	0.035
Mortality‐related bleeding complications	4 (6.3)	1 (2.0)	3 (21.4)	0.032
Hospital mortality	35 (55.6)	25 (51.0)	10 (71.4)	0.294

*Note*: The values represent medians, minimums and maximums or absolute and relative frequencies (%). The *p*‐value was obtained using the Mann–Whitney test, the Chi‐square test of independence for contingency tables or the Fisher's exact test.

Table 1: Description of the study group and outcomes.

Both groups were similar in terms of the initial severity of the illness (APACHE II score). In the AG group, patients stayed on ECMO longer with higher weaning success and lower in‐hospital mortality, but these results did not reach statistical significance. In the UFH group, only the alpha variant of COVID‐19 was present. In the AG group, the alpha variant was present in 53%, the delta variant in 43% and the omicron variant in 4%. Life‐threatening bleeding complications were significantly higher in the UFH group compared to the AG group (35.7% vs. 10.2%, *p* = 0.035) and also mortality‐related bleeding complications were higher in the UFH group (21.4% vs. 2.0%, *p* = 0.032).

**FIGURE 1:**
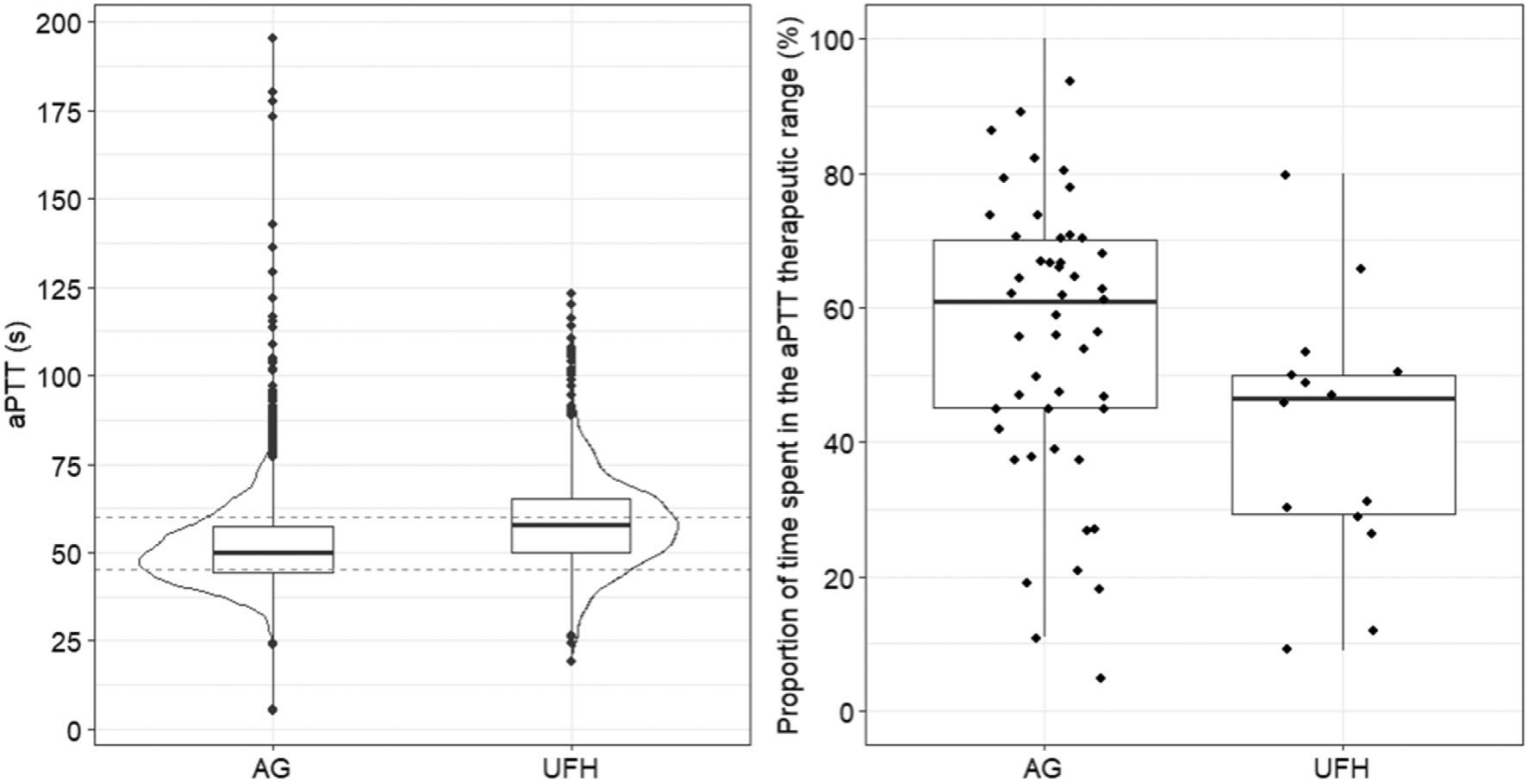
Visualisation of the distribution of all aPTT measurements (left; grey dashed lines show the aPTT therapeutic range) and the distributions of the proportion of time spent in the aPTT therapeutic range in individual patients (right) for the and UFH groups. AG, argatroban; UFH, unfractionated heparin.

Distributions of all aPTT measurements for the AG group and UFH group are compared in Figure 1 (left panel). The AG group showed a significantly lower median aPTT than the UFH group (50.1 vs. 57.7 s; Mann–Whitney test, *p* < 0.001); nevertheless, both medians were within the therapeutic range. The AG group also appeared to show significantly higher variability of aPTT values than the UFH group (Levene test, *p* < 0.001). However, when analysing intra‐individual variability of aPTT (i.e., the variability of aPTT in individual patients; right panel of Figure 1) using the robust coefficient of variation (RCV), patients in the AG group showed significantly lower RCVs (Median, Min‐Max: 8.1%, 2.4%–20.3%) than patients in the UFH group (10.9%, 5.6%–36.4%; Mann–Whitney test, *p* = 0.026), indicating that the intra‐individual variability of aPTT was lower in patients in the AG group. Furthermore, patients in the AG group showed a significantly higher proportion of time spent in the aPTT therapeutic range (61.0%, 5.0%–100.0%) compared to the UFH group (46.5%, 9.0%–80.0%); (Mann–Whitney test, *p* = 0.027).

**FIGURE 2:**
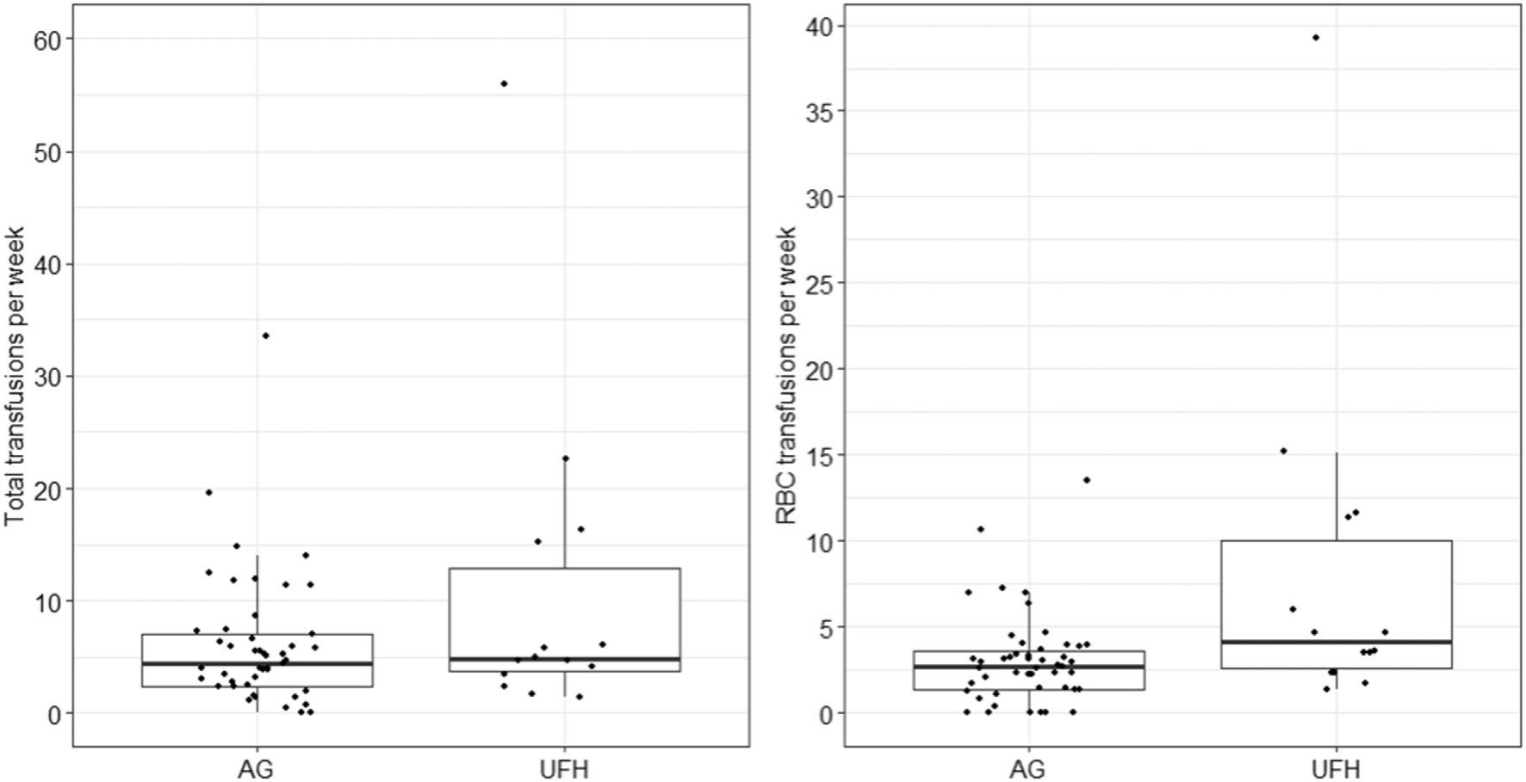
Distribution of the number of transfusions administered per week in individual patients in the AG and UFH groups. Left–insignificant difference for total transfusions (*p* = 0.275); right–significant difference in RBC transfusions (*p* = 0.011). AG, argatroban; UFH, unfractionated heparin.

The transfusions administered per week in each patient were determined, and the groups were compared. At least one transfusion of any type (red blood cell (RBC), platelets, and plasma) was administered to 89.8% (median total transfusions 4.4/week; min‐max 0.0–33.5/week) of patients in the AG group and 100% of patients in the UFH group (median 4.8/week; 1.4–56.0/week). This difference was not statistically significant (*p* = 0.275). The RBC consumption in the AG group (median 2.7 RBC/week; 0.0–13.5 RBC/week) was significantly lower than in the UFH group (median 4.2 RBC/week; 1.4–39.2 RBC/week, Mann–Whitney test, *p* = 0.011), see Figure 2. RBCs were administered to 87.8% of the AG group and 100% of the UFH group. No statistically significant difference was detected either in platelets transfusions that were administered to 57.1% (median 0.5 platelets unit/week; 0.0–8.5 platelets unit/week) of patients in the AG group and 50.0% of those in the UFH group (median 0.3 platelets unit/week; 0.0–9.8 platelets unit/week) or in plasma transfusions, which were administered to 46.9% (0.0–14.0 plasma unit/week) of patients in the AG group and 21.4% of those in the UFH group (0.0–7.0 plasma unit/week).

Two life‐threatening oxygenator thromboses occurred in the UFH group, while none were observed in the AG group. Over the 31 788 h of ECMO support (26 980 h in the AG group and 4808 h in the UFH group), 35 elective oxygenator changes were performed, of which 34 were in the AG group and one in the UFH group.

## 4 DISCUSSION

In our study, there was significantly less bleeding and ERD administered in the AG group compared to the UFH group. Also, life‐threatening bleeding occurred less in the AG group and this group also had fewer mortality‐related bleedings. Overall mortality was lower in the AG group, but not statistically significant. Overall, the mean time on ECMO was 16 days, which is comparable to data from the ELSO (Extracorporeal Life Support Organisation) registry, where the median time on ECMO for COVID‐19 was 14 days^9^ and review by Ramanathan et al. that is, 15 days.^10^

Anticoagulation with heparin is by far the most common anticoagulant used in ECMO. Despite some advantages, it can lead to specific complications in many cases. Martucci et al. describe anticoagulation management in a multicentre study of more than 600 patients.^6^ In 77%, UFH was the initial anticoagulant and aPTT was the most common test for monitoring (86%) with a median of 52 s. More than half experienced at least one bleeding event and 1.6% were fatal. UFH could be adjusted to lower anticoagulation targets to minimise the risk of bleeding.^5^ UFH is fundamentally involved in the homeostasis of coagulation by blocking free thrombin through interaction with antithrombin (AT). Besides, it also blocks other factors, such as fXa, fIXa, fXIa, the tissue factor/factor VIIa complex, induces tissue factor pathway inhibitor and is involved in several regulatory mechanisms in the organism.^11^, ^12^, ^13^ Heparin resistance (i.e., a situation when standard doses of UFH do not result in the expected values of the appropriate test) is caused by nonspecific binding of UFH to plasma proteins, high fibrinogen, and factor V levels or most commonly, by low levels of AT.^14^ Given the key role of the AT pathway in UFH action,^15^ it is obvious that a decrease in AT blood levels can reduce the UFH anticoagulation activity, thus increasing the risk of thrombotic events. On the other hand, supplementation of AT could lead to an unpredictable increase in the UFH effect, causing bleeding complications. Although high AT (>80%) demonstrated a higher platelet count and lower rate of platelets transfusions,^15^ in our cohort, the groups did not differ significantly in the number of platelets or plasma transfused. AT activity above 80% had higher survival rates.^15^ However, it is not clear whether this is related to the AT level itself or to the overall level of UFH anticoagulation. Therefore, there are no data on whether AT supplementation alone improves prognosis, for example in patients on anticoagulation other than UFH. It is also necessary to point out that heparin‐AT complexes are also ineffective when thrombin is already bound to fibrin. These factors, along with a high degree of UFH binding to plasma proteins, endothelial cells, and macrophages result in a lower predictability of UFH. This is particularly important from the perspective of binding to acute phase proteins as the change in their levels (e.g. during sepsis) affects the UFH action.^16^ UFH could also cause heparin‐induced thrombocytopenia^17^ with an up to 17% incidence in ECMO.^18^ The main advantage of UFH lies in the existence of its antagonist, protamine, which can completely reverse the anticoagulant effect of heparin. UFH was also reported to have an antiviral effect,^19^ including activity against COVID‐19.^20^

Unlike UFH, DTIs bind directly to thrombin and do not need any cofactors (no AT) for their effect. Their binding to plasma proteins is low, which eliminates the risk of HIT. Their effect is, therefore, more easily predicted than that of UFH.^21^, ^22^ Argatroban could be used in patients with a risk of HIT but also as a standard anticoagulant, depending on the particular center protocols.^23^ It binds reversibly to the catalytic site of both free thrombin and thrombin in the coagulum, thus inhibiting both soluble and fibrin‐bound thrombin. It has an approximately linearly dose‐dependent effect and a short circulating half‐life of about 40 min, making it relatively easy to control.^24^ AG is eliminated by the liver, so doses have to be reduced in patients with hepatic dysfunction.^25^ The advantages of AG over bivalirudin (another promising DTI) lie in its independence from renal dysfunction and prevention of thrombotic events even in circuit parts with low blood flow (cardiac chambers, oxygenator, etc.). aPTT is the most widely used method of anticoagulation monitoring in UFH as well as of AG use.^26^

In our study, the UFH and AG groups were in different time periods and therefore the groups differed in the COVID‐19 variant (see Table 1). Also, the coagulopathy associated with COVID‐19 may have changed due to immunity from natural infection and vaccination and the emergence of new COVID‐19 variants.^27^ The COVID‐19 variant differs in the degree of hypercoagulation and trombotic events, but our study mainly concerned with bleeding caused by anticoagulation. The delta variant had a higher risk of thrombotic complications compared to other variants (OR 1.36),^28^ but in our study, no life‐threatening oxygenator thrombosis occurred in the AG group, where the delta variant was present in 43% of patients. Elective circuit change was performed more frequently in the AG group, which may be related to the longer duration of patients on ECMO (12 vs. 18 days, *p* = 0.03).

According to ELSO 2021 anticoagulation guidelines,^29^ anticoagulation should be individualised according to a specific center protocol and no general standard exists. Physicians are very familiar with UFH use. So far, no data unanimously demonstrating the superiority of another anticoagulant over UFH in ECMO is available. Still, DTIs appear to be a promising alternative, especially thanks to the fact that their direct effect on thrombin is not influenced by other factors and that they appear to possess no clinically significant undesirable side effects. The aPTT is the most commonly used method of monitoring anticoagulation in both UFH and DTIs, although its value may be biased especially in proinflammatory states. In the study of Rad et al. COVID‐19 was associated with hyperinflammatory state in 67%, but only 19% of patients had concurrent coagulopathy.^30^ Some centers use for example antiXa or ROTEM, but in our protocol we used aPTT. Fisser et al. evaluated the outcomes of AG versus heparin anticoagulation in 117 ECMO patients without heparin‐induced thrombocytopenia. The rates of major bleeding and thrombotic complications in their study were comparable, with AG possibly having a smaller impact on the platelet decrease.^31^ Direct drug costs were higher for AG; however, when accounting for HIT testing and transfusions, the costs for UFH and AG were comparable. Cho et al. found similar results in the overall costs of patients treated with AG compared to those treated with UFH.^32^ In patients on ECMO due to respiratory failure caused by SARS‐CoV‐2 infection, Sattler et al. found a high prevalence of heparin resistance, making AG anticoagulation in these patients more manageable, without compromising safety.^33^ However, Stammers et al. reported that hepatic failure, renal failure and mortality were more common in DTI patients than in UFH patients.^34^

In our study, AG‐coagulated patients were relatively more often in the target aPTT range than UFH patients, which suggests that titrating the AG dose to the aPTT target range was more successful than in the case of UFH. The median aPTT in the UFH group was significantly higher than in the AG group, which might have led to a greater incidence of bleeding in the UFH group. Still, the median value of 57.7 s in the UFH group is within the target range considered safe (below 60 s). The greater incidence of bleeding (Life‐threatening bleeding complications and mortality‐related bleeding complications) in the UFH group is further confirmed by the overall greater need for transfusions in this group compared to the AG group. The difference between groups in all‐cause hospital mortality is not statistically significant. It should be noted that the higher incidence of bleeding complications in the UFH group not only results in a greater risk of potentially life‐threatening complications, but also in greater costs, greater consumption of valuable resources such as the ERD, and, last but not least, also in more demanding care for the patient. In the AG group, we have recorded no oxygenator thrombosis requiring an urgent oxygenator exchange, while in the UFH group, two such events were recorded (even though the total time on ECMO was more than six times higher in the AG group than in the UFH group).

## 5 LIMITATIONS

The main limitation of our study is the small sample size in both groups and uneven group sizes. Moreover, the UFH group and AG group were recruited in different periods, implying that different predominant SARS‐CoV‐2 strains in the respective group might influence the severity and course of the disease, as well as the hemostatic status of the patients. Despite this, the two groups were comparable in APACHE II score and there was no difference in treatment management between the two groups. During the bleeding event, no single treatment protocol was used and the patient continued to be monitored. This could theoretically affect any further bleeding.

## 6 CONCLUSIONS

Our results suggest that AG has more stable aPTT levels and fewer bleeding complications compared to UFH, including significantly fewer life‐threatening and mortality‐related bleeds. ECMO support with AG anticoagulation was also feasible and safe concerning oxygenator thrombotic complications.

## AUTHOR CONTRIBUTIONS

**F. B:** Conceptualization; original draft preparation; approval of the final text. **J. M:** Review & editing, approval of the final text; **J. S:** dataset assembly, starvation of COVID‐19 variant. **M. F:** Conducting the research and investigation process; approval of the final text.**P. S:** Supervision; critical revision of the manuscript; approval of the final text. **S. N:** Digitalization of the dataset; approval of the final text. **Z. K:** Digitalization of the dataset, approval of the final text. **O. J:** Conducting the research and investigation process; approval of the final text. **T. R:** Digitalization of the dataset; approval of the final text. **A. K:** Statistical analysis; approval of the final text. **J. J:** Statistical analysis; critical review and editing of the original draft; approval of the final text.

## FUNDING INFORMATION

This work and the contributions were supported by the Ministry of Health, Czech Republic–conceptual development of research organisation (FNOs/2024).

## CONFLICT OF INTEREST STATEMENT

The authors declare no conflicts of interest.

## Data Availability

The data are available upon reasonable request.
